# Almonds and Cardiovascular Health: A Review

**DOI:** 10.3390/nu10040468

**Published:** 2018-04-11

**Authors:** Soumik Kalita, Shweta Khandelwal, Jagmeet Madan, Himanshu Pandya, Boindala Sesikeran, Kamala Krishnaswamy

**Affiliations:** 1 FamPhy, Gurgaon 122101, India; 2 Public Health Foundation of India, Gurgaon 122001, India; shweta.khandelwal@phfi.org; 3 Sir Vithaldas Thackersey College of Home Science, SNDT Women’s University, Mumbai 400049, India; dr.jagmeetmadan@gmail.com; 4 Pramukhswami Medical College, Karamsad, Gujarat 388325, India; himanshuvpandya@gmail.com; 5 Former Directors National Institute of Nutrition, Hyderabad 500007, India; sesikeran@gmail.com (B.S.); sri21kk@yahoo.com (K.K.)

**Keywords:** almonds, lipids, heart disease, cardiovascular disease, nuts, dyslipidemia cholesterol, low density lipoprotein, high density lipoprotein

## Abstract

Several preventive strategies to reduce dyslipidemia have been suggested, of which dietary modification features as an important one. Dyslipidemia is a major risk factor for coronary heart disease and strategies to manage dyslipidemia have been shown to reduce the incidence of cardiovascular disease (CVD). Although there are proven pharmacological therapies to help manage this condition, nutritional interventions are a safer option to help prevent and manage dyslipidemia. Addition of almonds in the daily diet has been proposed to beneficially impact the lipid profile. This review critically examines the available evidence assessing the effect of almonds on dyslipidemia in the South Asian (particularly Indian) context. An extensive review comprised of epidemiological studies, clinical trials, meta-analyses, and systematic reviews was conducted from published literature from across the world. Studies examining the effect of almonds on different aspects of dyslipidemia viz. high low-density lipoprotein-cholesterol (LDL-C), low high-density lipoprotein-cholesterol (HDL-C), triglyceridaemia, and high total cholesterol levels have been included. In several studies, almonds have been shown to reduce LDL-C—which is a known risk factor for CHD—and the effect of almonds has been well documented in systematic reviews and meta-analysis of clinical trials. Addition of almonds in the diet has been shown to not only to reduce LDL-C levels, but also to maintain HDL-C levels. This review provides information about the use of this simple nutritional strategy which may help manage known major risk factors for heart disease, such as high LDL-C and low HDL-C levels especially in the context of South Asians.

## 1. Introduction

India, like many other developing nations in the world, has been facing the dual burden of malnutrition with undernutrition on one hand and diet related chronic diseases on the other. There are millions of people who suffer from undernutrition whereas there is a huge emerging population with effects of overnutrition like obesity with its related issues. Of all the chronic diseases, cardiovascular diseases (CVD) appear to be a major public health concern and warrants the attention of public health scientists, clinicians, and policy makers. Although pharmacological approaches have been in practice, life style modifications—particularly diet, physical activity, and smoking cessation—are amongst the best preventive strategies.

Indians have a higher risk for heart diseases owing to their genetic make-up and this has been documented in several epidemiological studies [1]. The theory of Indians having a lower threshold for risk factors for heart disease due to their genetic makeup can be applicable to the large Indian diaspora across the globe, which has been estimated to be around 31 million and to be the largest in the world [2]. Coupled with lifestyle issues—like lack of physical activity and food rich in sugars, salt, and saturated fats—it makes Indians prone to heart diseases much more than their Caucasian counterparts. Cardiovascular diseases account for 28% of total mortality in India surpassing all other causes [3]. The age-standardized death rate from CVD is 272 per 100,000 populations in India in comparison to the global average of 235 [4]. The impact of CVDs as measured by the years of lives lost to the disease, was 37 million in the year 2010, rising from 23.2 million (nearly 60% more) in just 20 years [4]. Risk factors for CVDs—especially coronary heart disease (CHD)—like high LDL cholesterol (LDL-C), low HDL cholesterol (HDL-C), and abnormal apolipoprotein A-1:B ratio, are increasing at a steady pace. It is essential to prevent further rise in its incidence and prevalence [5]. Dyslipidemia can be clinically defined as elevated total cholesterol or LDL-C levels and a low HDL-C levels. Indians are at a higher risk of CHD due to changing lifestyles resulting in an increasing number of people with conditions like abdominal obesity (visceral adiposity), insulin resistance, dyslipidemia, and inflammation [6]. Increases in the apolipoprotein B100/apolipoprotein A-I ratio is more prevalent in South Asians (43.8%) than people from other geographies (31.8%) [7]. This is a major risk factor, out of the eight risks factors, that explains more than 90% of deaths due to myocardial infarction (MI) amongst Indians [8].

The INTERHEART study, which was conducted in 52 countries, including India, mentions dyslipidemia as a potentially modifiable risk factor for MI [8]. The secular trend seen in the rise of CVDs in India is also due to the consumption of saturated fats, trans-fats, refined carbohydrates, and processed foods among Indians [9]. The prevalence of low HDL-C and hypertriglyceridemia are associated with coronary artery disease (CAD) in Indian patients [10]. In a large study conducted by the Indian Council of Medical Research (ICMR) in four regions of India, 79% of the participants were found to have some form of dyslipidemia. The ICMR study also identified that 72.3% of the total participants had low HDL-C levels, 29.5% had hypertriglyceridemia, and almost 12% had high LDL-C levels [11].

Strategies to manage dyslipidemia have been shown to reduce CVD in several studies. Systematic reviews and meta-analyses of several clinical trials show the reduction in LDL-C levels are directly proportional to the reduction in the CVD risk [12]. All the studies in these systematic reviews or meta-analyses looked at pharmacological therapies that help to lower LDL-C levels, of which statins were prominent. Notwithstanding the benefits of statins in reducing LDL-C levels in dyslipidemia, the use of this class of drugs also leads to adverse effects like myalgia [13]. A study called the Prediction of Muscular Risk in Observational Conditions (PRIMO) project, in which a cohort of 7924 hyperlipidemic patients received high-dose statin therapy, 10.5% of patients reported myalgia like symptoms [13]. A meta-analysis of 90 observational, cross-sectional, and cohort studies found a strong positive association (OR: 2.63; 95% CI; 1.50–4.61) of statins and myopathy [14].

Systematic reviews document a special role for lifestyle modification on the prevention of CVD and its risk factors and therefore these need to be emphasized as part of strategies to prevent or manage CVD [15]. Nutritional interventions to help manage dyslipidemia by mainly reducing LDL-C levels and increasing HDL-C levels have to be a part of lifestyle modifications along with enhanced physical activity levels. Changing dietary habits by reducing sugars, saturated fats, and salts in the population has potential to help people to prevent as well as manage lifestyle diseases like coronary heart disease and therefore nutritional intervention plays a major role in this pursuit.

Prospective studies on nut consumption on a regular basis have shown reduction in the risk of heart disease as well as mortality in cohort studies [16,17]. In a large systematic analysis for the Global Burden of Disease study, low consumption of nuts and seeds as risk factors for cardiovascular and circulatory diseases was observed [18]. There was an inverse association seen between nut consumption and sudden cardiac deaths in the prospective data of 21,454 male participants in the US Physicians’ Health Study [17]. Incorporation of nuts, especially almonds, in the diet of Americans has been shown to improve nutrient quality in both children and adults and could be adopted as a strategy to replace unhealthy snacks [19,20]. Consumption of almonds has been shown to have positive effects on health, especially in the area of metabolic disorders [21].

Almonds have been a part of the traditional Indian diet since time immemorial and have been a part of several Indian cuisines. Traditionally, almonds in India are soaked and peeled to be eaten early in the morning and has had several positive connotations with respect to health. Almonds are rich in nutrients like vitamin E, proteins, Mono-unsaturated fatty acids (MUFAs), Polyunsaturated fatty acids (PUFAs), magnesium, potassium, and dietary fibers, which are all useful for good cardiovascular health. There is strong clinical evidence that proves the beneficial effect of almonds in dyslipidemia management. Several well conducted studies from across the world have shown that almonds have a potential to reduce cardiovascular risk factors like dyslipidemia, namely high LDL-C and low HDL-C levels.

## 2. Almonds and Dyslipidemia

Almonds are a part of the prunus family, which are rich sources of mono- and polyunsaturated fatty acids (MUFAs and PUFAs) and are widely accepted in India as a nutritive food for several benefits [22]. A portion of 100 g of almonds contain around 50 g of healthy fats, most of which (40 g) are MUFAs and PUFAs, along with 4 g of saturated fats [23]. Almonds are a rich source of several minerals as well as vitamins like calcium, copper, iron, magnesium, phosphorus, potassium, zinc, manganese, thiamine, riboflavin, niacin, and vitamin E. The health benefits of almonds can be attributed to their healthy fatty acid composition, high vitamin E and fiber content, as well as other nutrients (Table 1). Almond skin flavonoids have been shown to possess anti-oxidant activity in in vitro models, to be bioavailable and to synergistically act along with vitamin E to prevent oxidation of LDL in hamster models [24]. Almonds are rich in lipids but the bio-accessibility of lipids from almonds has been shown to be poor in different studies which could be attributed to the cell wall of almonds, which is rich in non-starch polysaccharides, thus preventing or reducing lipid release [25]. The way almonds are consumed determines the measured metabolizable energy (ME). The measured ME of whole natural almonds, whole roasted almonds and chopped almonds were shown to be significantly less than the measured ME of almond butter, whereas the ME of whole natural almonds were lesser than that of whole roasted almonds [26].

This review captures an in-depth analysis of effect of almonds on managing lipid levels, thus providing clinical evidence for mitigating risk factor of dyslipidemia, one of the major drivers of CVD in Indians.

## 3. Almonds and LDL-C Levels

A meta-analysis observed a significant reduction in LDL-C levels with almond consumption [27]. Almonds have been shown to have a dose-dependent effect on the reducing LDL-C in several well conducted studies [28,29,30]. A recent systematic review found that eating almonds results in significant reductions in total cholesterol, LDL-C, and triglycerides levels, while having no significant impact on HDL-C levels [30]. A sub-group analysis showed that blood lipid levels substantially improved in the studies in which the dose of almonds was at least 45 g/day in individuals with altered lipid profiles. The analysis which included 18 published randomized controlled trials (selected from a pool of 1697 publications) and a total of 837 participants showed the reduction in total cholesterol by 0.153 mmol/L (5.92 mg/dL). In yet another sub-group analysis of studies where the amount of almonds consumed was at least 45 g/day (~1.5 oz/day), the reduction in total cholesterol was 0.212 mmol/L (8.20 mg/dL) which suggested that the effects of almonds on total cholesterol are dose-dependent. A similar pattern was observed for LDL-C with the calculated reduction of 0.124 mmol/L (4.80 mg/dL) in the pooled data and reductions of 0.132 mmol/L (5.10 mg/dL) after a sub-group analyses of those studies in which at least 45 g (~1.5 oz) of almonds were consumed per day [31].

The effect of almond consumption on lipids was seen in several studies, one of earliest of which was published in 1992 in which the diet of the participants was modified by adding 100 g of almonds to it [32]. There was a reduction in the total plasma cholesterol levels by 21 mg/dL (*p* < 0.05) in a period of nine weeks. Several studies followed the study done by Spiller et al., 1992 which showed similar results of almonds on the reduction of cholesterol and LDL-C levels [28,30,33,34,35,36,37,38]. In a recently concluded large randomized controlled crossover study, conducted over a period of six weeks, the consumption of almost 45 g of almonds (1.5 ounces) reduced LDL-C levels and non-HDL-C levels significantly while maintaining HDL-C levels [39]. The study also demonstrated that almond intake reduced abdominal fat which is known to be a major factor in metabolic syndrome and ischemic heart disease (IHD). A study from India in subjects with metabolic syndrome demonstrated a significant decrease in LDL-C and total cholesterol levels after consumption of 30 g of almonds for a period of six weeks [40]. The randomized controlled study also showed statistically significant reduction in waist circumference, body mass index (BMI), and systolic blood pressure in the group that consumed almonds as part of the intervention. In another recent study from India, almonds were shown to reduce LDL-C, serum triglycerides, and cholesterol levels along with markers of metabolic syndrome—such as glycosylated hemoglobin and abdominal obesity in patients with type 2 diabetes [41]. The intervention duration in this study was 24 weeks during which 20% of the participant energy intake was replaced with almonds.

A theoretical modelling study compared the effects of relative almond intake (defined as almonds consumed per body weight) and that of reduction of dietary saturated fats alone. The study observed that increased almond intake was more beneficial than the reduction of dietary saturated alone and that replacing saturated fats with almonds on a regular basis could help reduce LDL-C levels further [42].

## 4. Almonds and HDL-C Levels

Low HDL-C is a key CVD risk factor as seen in several epidemiological studies [43,44]. Low HDL-C levels have been found to be prevalent in people with insulin resistance even without the presence of type 2 diabetes mellitus [45]. A large multicenter study done in India estimated that nearly 72% of Indians have low HDL-C levels [11]. According to The Indian Heart Association, every 10-point increase in HDL-C may reduce the risk of heart disease by half [46]. Physicians worldwide are intrigued as well as find it difficult to enhance HDL-C levels even after prescribing strict exercise regimes and the addition of pharmacological therapies like high doses of niacin, fibric acids, or bile acid sequestrants. Adult Treatment Panel III (ATP III) guidelines increased the healthy HDL-C threshold from less than 35 mg/dL to less than 40 mg/dL in males and to 50 mg/dL in females [47]. The American Association of Clinical Endocrinologists and American College of Endocrinology guidelines for management of dyslipidemia and prevention of cardiovascular disease in its 2017 guidelines recommends a minimum HDL-C level of 40 mg/dL in individuals with dyslipidemia and atherosclerotic cardiovascular disease risk [48].

One of paradoxes of lifestyle interventions is that dietary strategies for reducing LDL-C and cholesterol levels like reduction of dietary saturated fats has also been shown to reduce HDL-C levels [49]. Therefore, it is important for maintaining or even increasing HDL-C levels while lowering LDL-C levels. A recent randomized controlled study conducted in Pakistan investigated the effect of almonds on HDL-C levels in patients suffering from coronary artery disease (CAD). This is a seminal study that tried to look at the effects of almonds exclusively in a South Asian population. The study assessed 1489 patients suffering from CAD for recruitment eligibility, from which 150 subjects were recruited into the study. Of these, 113 were men and 37 were women. The study showed that almonds significantly increase HDL-C levels up to an average of 14% in patients with CAD in just six weeks. The participants were given a daily dose of 10 g of soaked and peeled almonds as an intervention, which is also a traditional way of consuming almonds in India [49].

A study done in New Zealand, examined the effect of nuts (almonds and hazelnuts) in different forms (ground, sliced, and whole) on the HDL-C levels after six weeks of intervention and found a statistically significant reduction in LDL-C levels along with an increase in HDL-C levels [50].

Recent literature mentions that absolute HDL-C levels alone may not be cardioprotective but this effect may be dependent on the HDL-C functions as well as the sub-species of HDL-C e.g., plasma apoA-I–containing HDL-C subspecies [51]. In a recent study, consumption of 43 g of almonds every day over six weeks helped elevate HDL-C levels in the intervention group in comparison to the control group [52].

The above-mentioned studies have been able to demonstrate significant improvement in HDL-C levels, thus suggesting a viable dietary approach for management of an important CVD risk factor.

## 5. Almonds and Other Lipid Parameters

Hypertriglyceridemia is an important risk factor for CVD, especially in the context of rising trends of obesity and insulin resistance worldwide [53]. An analysis of 101 studies has found a causal relationship between hypertriglyceridemia and CAD [54]. South Asians have been shown to have a pattern of dyslipidemia which is different from people of other ethnicity which could be one of the reasons for the early propensity for coronary heart disease in this population. This pattern of dyslipidemia in South Asians consists of low HLD-C levels, increased lipoprotein-A levels, as well and increased number of atherogenic particles in comparison to people of other origins with similar LDL-C levels [55]. South Asians have been shown to have lower HDL-C and higher triglyceride levels in comparison to their Caucasian counterparts [56].

Although a study conducted in a population suffering from CAD has shown the effect of almonds in reducing the levels of triglycerides in people with CAD at 6 and 12 weeks of intervention with 10 g of almonds (*p* < 0.05) [49], more studies need to be conducted to further investigate this effect. New biomarkers, like LDL particle number and LDL particle size, have been found to be associated with CVD with some studies suggesting these markers to be more sensitive than LDL-C or HDL-C [57]. There is a need for research on the effects of almonds not only on the conventional biomarkers for CVD but also for newer ones like LDL particle size and number.

Addition of almonds to a lifestyle modification model along with increased physical activity can be a natural way to prevent and manage cardiovascular disease risk factors, particularly dyslipidemia, which is a major concern for the Indian population.

## 6. Conclusions

Almonds have been shown to reduce LDL-C, which is a known risk factor for coronary heart disease in several well conducted clinical trials. Studies have also looked at the effect of almonds on HDL-C and it has been found that consumption of almonds have helped maintain or even increase HDL-C levels. Daily consumption of around 45 g of almonds can help reduce one the most important risk factors for CVD in Indians, viz. dyslipidemia. Addition of whole almonds in the diet is a safe and practical nutritional strategy that can be recommended to manage dyslipidemia. Further studies need to be conducted to investigate the mechanism of action responsible for the effect of almonds on dyslipidemia as well as its effect on lipid particle size and vascular health.

## Figures and Tables

**Table 1:** Almond composition.

Nutrients	Units	Value per 100 g Whole Almonds
Proximates		
Calories	kcal	579
Water	g	4.41
Protein	g	21.15
Lipids (total)	g	49.93
Dietary fiber (Total)	g	12.5
Sugars (Total)	g	4.35
Ash	g	2.97
Minerals		
Calcium	mg	269
Iron	mg	3.71
Magnesium	mg	270
Phosphorus	mg	481
Potassium	mg	733
Sodium	mg	1
Zinc	mg	3.12
Copper	mg	1.03
Manganese	mg	2.18
Vitamins		
Vitamin E (alpha-tocopherol)	mg	25.63
Thiamin	mg	0.21
Riboflavin	mg	1.14
Niacin	mg	3.62
Pantothenic acid	mg	0.47
Vitamin B6	mg	0.14
Folate, food	mcg	44
Fatty Acids		
Saturated (TOTAL)	g	3.80
16:0 Palmitic	g	3.08
18:0 Stearic	g	0.70
Monounsaturated (total)	g	31.55
16:1 Palmitoleic	g	0.23
18:1 Oleic	g	31.29
Polyunsaturated (total)	g	12.33
18:2 Linoleic	g	12.32

USDA 2016 SR28 Nutrient Database No.12061 Nuts, almonds.

## Abbreviations

CVD	Cardiovascular disease
CAD	Coronary artery disease
LDL-C	Low density lipoprotein-cholesterol
HDL-C	High density lipoprotein-cholesterol
MUFA	Monounsaturated fatty acid
PUFA	Polyunsaturated fatty acid
